# Supplementary material for: Impact of a Dedicated Pretransplant Infectious Disease Consultation on Respiratory Tract Infections in Kidney Allograft Recipients: A Retrospective Study of 516 Recipients
Source: Pathogens. 2023 Jan 3;12(1):74. doi: 10.3390/pathogens12010074 (PMC9867402; doi:10.3390/pathogens12010074)
Supplement: Supplementary file 1 [file pathogens-12-00074-s001.zip › pathogens-2074063-supplementary.pdf]

Supplementary Table S1. Respiratory tract infection risk and protective factors: detailed results from univariate logistic regression model.

| Variables | OR | 95% CI | p-value |
|---|---|---|---|
| **Recipient characteristics** | | | |
| Female | 1.06 | 0.67-1.67 | 0.81 |
| Age at transplantation | 1.02 | 1.003-1.04 | **0.019** |
| Dialysis | 0.77 | 0.41-1.43 | 0.41 |
| HIV + | 4.51 | 2.21-9.23 | **< 0.0001** |
| HCV + | 6.34 | 2.93-13.70 | **< 0.0001** |
| CMV + | 1.64 | 0.87-3.08 | 0.13 |
| **Initial nephropathy** | | | |
| Hypertension | 1.71 | 0.82-3.54 | 0.15 |
| Unknown origin | 0.79 | 0.46-1.37 | 0.41 |
| Diabetes | 1.24 | 0.69-2.23 | 0.48 |
| Genetic | 0.2 | 0.06-0.67 | **0.008** |
| Glomerulopathy | 0.81 | 0.48-1.38 | 0.44 |
| Tubular and interstitial | 0.57 | 0.2-1.67 | 0.31 |
| Obstructive | 2.26 | 0.55-9.18 | 0.26 |
| Other | 3.61 | 1.85-7.02 | **0.0002** |
| **Donor characteristics** | | | |
| Age | 1.01 | 1.00-1.03 | 0.12 |
| Creatinine > 100 umol/L | 0.83 | 0.49-1.43 | 0.51 |
| Living donor | 0.62 | 0.31-1.26 | 0.19 |
| Extended Criteria Donor | 1.47 | 0.94-2.31 | 0.19 |
| **Infection disease consultation before transplant** | 0.5 | 0.28-0.88 | **0.016** |
| **Combined transplants** | | | |
| Liver | 2.65 | 1.13-6.19 | **0.02** |
| Pancreas | 4.51 | 0.63-32.4 | **0.01** |
| Heart | 0.89 | 0.10-7.66 | 0.91 |
| **Kidney transplant characteristics** | | | |
| Previous transplant | 1.29 | 0.67-2.5 | 0.45 |
| Cold ischemia time | 1 | 0.97-1.03 | 0.81 |
| Induction immunosuppressive regimen | | | |
| Basiliximab | 1.02 | 0.64-1.63 | 0.94 |
| Antithymocyte globulin | 1.06 | 0.66-1.69 | 0.82 |
| Intraveinous imunoglobulin | 0.78 | 0.38-1.60 | 0.51 |
| Rituximab | 1.25 | 0.60-2.62 | 0.56 |
| Maintenance immunosuppressive regimen | | | |
| Ciclosporine | 0.78 | 0.29-2.07 | 0.62 |
| Tacrolimus | 0.82 | 0.43-1.55 | 0.54 |

| | | | |
|---|---|---|---|
| Mycophenolate mofetil | 0.82 | 0.50-1.36 | 0.44 |
| Steroids | 2.04 | 0.08-54.01 | 0.34 |
| Belatacept | 1.74 | 0.84-3.63 | 0.14 |
| Certican | 1.3 | 0.78-2.15 | 0.32 |

*OR: odds ratio from unadjusted logisitc regression model. HIV, Human Immundeficiency Virus; HCV, Hepatitis C Virus; CMV, Cytomegalovirus.* ***Bolded*** *results are statistically significant at the p<0.05 level.*
